# Large language models management of complex medication regimens: a case-based evaluation

**DOI:** 10.3389/fphar.2025.1514445

**Published:** 2025-11-24

**Authors:** Aaron Chase, Amoreena Most, Shaochen Xu, Erin Barreto, Brian Murray, Kelli Henry, Susan Smith, Tanner Hedrick, Xianyan Chen, Sheng Li, Tianming Liu, Andrea Sikora

**Affiliations:** 1 Department of Pharmacy, Wellstar MCG Health, Augusta, GA, United States; 2 Department of Pharmacy, UNM Health System, Albuquerque, NM, United States; 3 Department of Computer Science, University of Georgia, Athens, GA, United States; 4 Department of Pharmacy, Mayo Clinic, Rochester, MN, United States; 5 Department of Clinical Pharmacy, University of Colorado Skaggs School of Pharmacy, Aurora, CO, United States; 6 Department of Clinical and Administrative Pharmacy, University of Georgia College of Pharmacy, Athens, GA, United States; 7 Department of Pharmacy, University of North Carolina Medical Center, Chapel Hill, NC, United States; 8 Department of Epidemiology & Biostatistics, University of Georgia College of Public Health, Athens, GA, United States; 9 School of Data Science, University of Virginia, Charlottesville, VA, United States; 10 Department of Biomedical Informatics, University of Colorado School of Medicine, Aurora, CO, United States; 11 Department of Clinical and Administrative Pharmacy, University of Georgia College of Pharmacy, Augusta, GA, United States

**Keywords:** large language model, artificial intelligence, pharmacy, medication regimen complexity, natural language processing (NLP)

## Abstract

**Background:**

Large language models (LLMs) have shown the ability to diagnose complex medical cases, but only limited studies have evaluated the performance of LLMs in the development of evidence-based treatment plans. The purpose of this evaluation was to test four LLMs on their ability to develop safe and efficacious treatment plans on complex patients managed in the intensive care unit (ICU).

**Methods:**

Eight high-fidelity patient cases focusing on medication management were developed by critical care clinicians including history of present illness, laboratory values, vital signs, home medications, and current medications. Four LLMs [ChatGPT (GPT-3.5), ChatGPT (GPT-4), Claude-2, and Llama-2–70b] were prompted to develop an optimized medication regimen for each case. LLM generated medication regimens were then reviewed by a panel of seven critical care clinicians to assess safety and efficacy, as defined by medication errors identified and appropriate treatment for the clinical conditions. Appropriate treatment was measured by the average rate of clinician agreement to continue each medication in the regimen and compared using analysis of variance (ANOVA).

**Results:**

Clinicians identified a median of 4.1–6.9 medication errors per recommended regimen, and life-threatening medication recommendations were present in 16.3%–57.1% of the regimens, depending on LLM. Clinicians continued LLM-recommended medications at a rate of 54.6%–67.3%, with GPT-4 having the highest rate of medication continuation among all LLMs tested (p < 0.001) and the lowest rate of life-threatening medication errors (p < 0.001).

**Conclusion:**

Caution is warranted using present LLMs for medication regimens given the number of medication errors that were identified in this pilot study. However, LLMs did demonstrate potential to serve as clinical decision support for the management of complex medication regimens given the need for domain specific prompting and testing.

## Introduction

Large language models (LLMs) have demonstrated proficiency across a wide spectrum of natural language processing (NLP) tasks, including notable achievements like passing medical licensing exams and making correct diagnoses of complex patient cases (Kanjee et al., 2023; Gilson et al., 2023). However, these tasks have largely focused on highly structured problems of disease diagnosis, and LLMs have undergone limited evaluations for the more unstructured task of choosing the correct treatment course for the diagnosed disease (Bužančić et al., 2024; Hsu et al., 2023; Kunitsu, 2023).

Comprehensive medication management (CMM) refers to “the standard of care that ensures each patient’s medications are appropriate, effective for the medical condition, safe given the comorbidities and other medications being taken, and able to be taken as intended.” (ASHP, 2025) Each year, there are approximately 1.8 million adverse drug events (ADEs) in hospitalized patients with estimates that 9,000 patients die as a direct result of a medication error (Leape et al., 1999; Nuckols et al., 2014; Slight et al., 2018). Costs related to medication errors exceed $40 billion (Tariq et al., 2024). Given the morbidity and cost to the healthcare system associated with ADEs, evaluating novel tools such as LLMs for the potential to facilitate CMM activities and improve medication safety is essential (Kwan et al., 2025). LLMs process text and understand human language in large quantities and at rapid speeds, which can be helpful in fields such as healthcare and medication management, which include large amount of information processing (Kwan et al., 2025). Thus far, LLMs have been tested specifically in the realm of medication management for deprescribing benzodiazepines, identifying drug-herb interactions, and performance on a national pharmacist examination (Bužančić et al., 2024; Hsu et al., 2023; Kunitsu, 2023). However, there have been no investigations for the potential for LLMs to aid in delivery of CMM.

The purpose of this pilot study was to compare performance of four LLMs [ChatGPT (GPT-3.5), ChatGPT (GPT-4), Claude-2, and Llama-2–70b] in conducting CMM for complex medication regimens for critically ill patients.

## Methods

### Study design

The primary objective was to evaluate the capabilities of LLMs in generating safe and efficacious treatment plans for complex patient cases. This involved a carefully structured prompting process, intended to elicit the most accurate and clinically relevant responses from the LLMs. Our study used a comparative analysis approach, testing four advanced LLMs: GPT-3.5, GPT-4, Llama-2–70b, and Claude-2. These LLMs were chosen to parallel other exploratory analyses by our team and were thought to be representative of LLM capability and functionality (Chase et al., 2025; Yang et al., 2024). Seven distinct patient cases were used in the fall of 2023, with one that served as an initial example for single-shot prompting, and the subsequent seven cases utilized as actual test scenarios. All test scenarios were entered in separate chats. ChatGPT was accessed via the chatbot interface using the standard settings of temperature = 0.7 and Top P = 1.0. Llama-2–70b was also used with the standard settings. The primary outcomes were based on the safety and efficacy of the recommended scenarios, as assessed by a panel of seven critical care clinicians. Safety was measured by the rate of clinician-identified medication errors and life-threatening medication errors recommended by the LLMs. Efficacy was measured by the average rate of clinician continuation of medications recommended by the LLMs. Other outcomes included the overall agreement of clinicians with the recommended regimen based on a five-point Likert scale and characterization of reasons for discontinuation of medications recommended by LLM.

### LLM testing

A total of eight patient cases were developed by critical care clinicians, with one used as an example in the prompting process. These patient cases included traditional critical care disease states, including sepsis, pneumonia, shock, diabetes, etc. Medication-related problems were intended to reflect critically ill patients cared for in the intensive care unit (ICU), and included evaluations for gastrointestinal ulcer prophylaxis, venous thromboembolism prophylaxis, antibiotic selection, sepsis management, etc. Cases incorporated a history of present illness, relevant laboratory and vital sign data, home medications, and current medications. The patient cases included a “ground truth” which was a list of appropriate medications determined to be the most correct approach to their management by the panel of clinicians, which was agreed upon via majority vote prior to LLM testing. The ground truth was provided to the LLM in the initial prompting process but then was asked to be generated by the LLM in the new patient scenario process. The approach employed a one-shot prompting with in-context learning designed to guide the LLMs through a structured evaluation of the patient cases to generate an optimized medication regimen (Holmes et al., 2023). This approach is especially beneficial in complex decision-making tasks, such as medical treatment planning, where contextual understanding and synthesis of information are crucial.

### One-shot prompting with in-context learning process

1. Initial Example Prompting: “Please review the case below and pay close attention to how the ground truth section at the end is structured.” This step involved providing the LLMs with a comprehensive patient case, including detailed medical history, current treatment plans, and the ground truth medication plan. The LLMs were instructed to closely analyze the structure and formatting of the ground truth section, which outlined the updated medication plan. This initial example served as a form of single-shot prompting, aiming to familiarize the LLMs with the expected output format and clinical reasoning required for generating appropriate medication plans.
2. New Patient Scenario Prompting: “Now, I will give you a separate case, please review all the information given and based on it provide a new updated prescribed medication list exactly like how the ground truth section is structured and formatted in the example given before.” Following the initial example, the LLMs were presented with new patient scenarios, each featuring unique conditions, clinical scenarios, and medications challenges. The LLMs were tasked with synthesizing this information to propose an updated medication plan, mirroring the structure and format of the ground truth example provided earlier.

**FIGURE 1:**
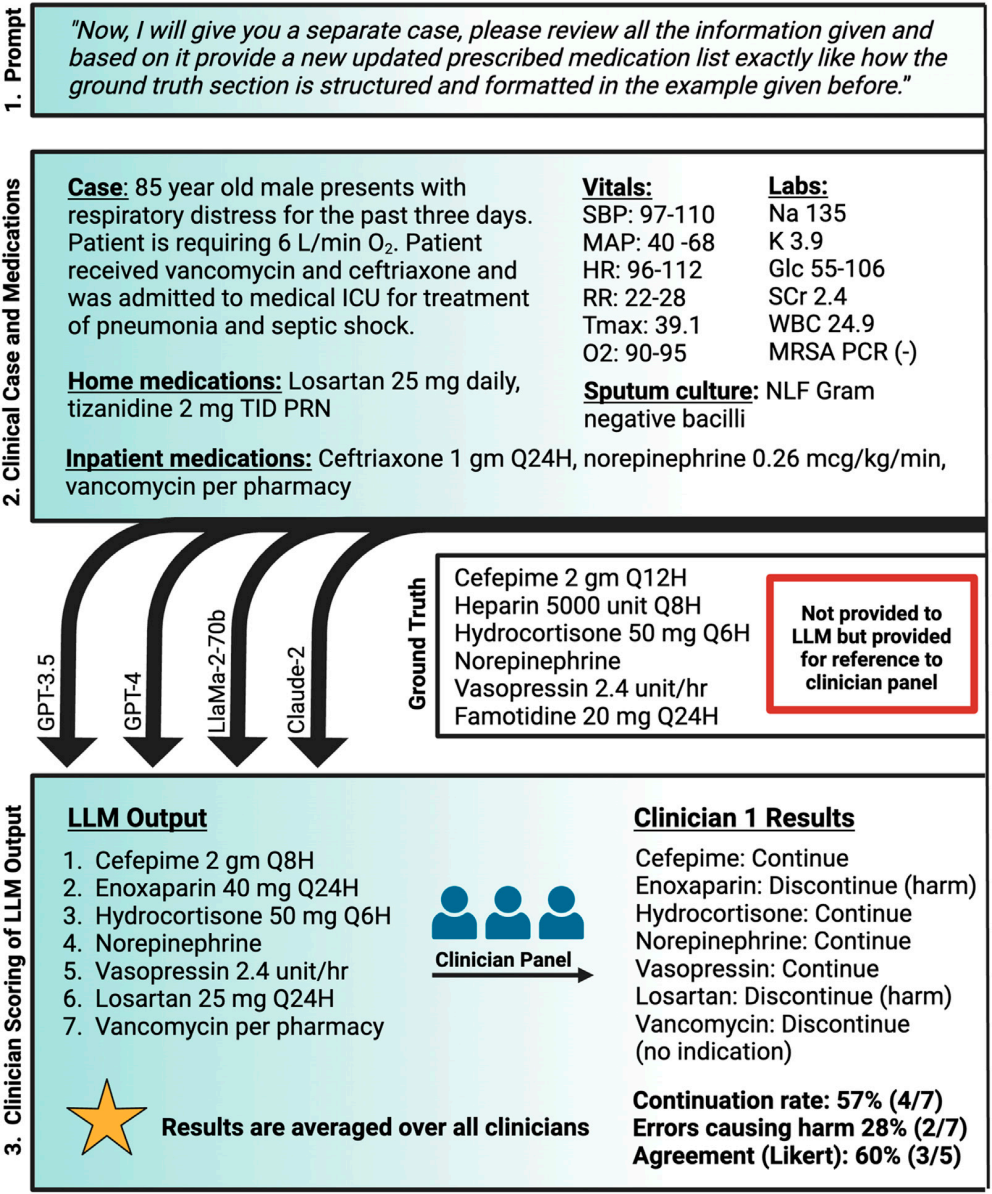
Methodology for LLM-assessment of comprehensive medication management Created with biorender.com. O2, oxygen; Glc, serum glucose; gm, Gram; HR, heart rate; ICU, intensive care unit; K, serum potassium; L/min, liters per minute; LLM, large language model; MAP, mean arterial pressure; mcg/kg/min, microgram per kilogram per minute; mg, milligram; MRSA PCR, methicillin-resistant *staphylococcus aureus* nasal polymerase chain reaction; Na, serum sodium; NLF, non-lactose fermenting; PRN, as needed; Q12H, every 12 h; Q24H, every 24 h; Q6H, every 6 h; Q8H, every 8 h; RR, respiratory rate; SBP, systolic blood pressure; SCr, serum creatinine; TID, three times daily; Tmax, maximum temperature; unit/hr, unit per hour; WBC, white blood cell.

A panel of seven critical care board-certified and critical care residency trained pharmacists was then asked to review the medication regimen generated by each of the four LLMs for the 7 test patient cases. Individuals were blinded to model identity and to each other. Each individual was asked to review the generated medication regimen and provide the following information: (1) itemized “continue” or “discontinue” recommendations for each medication in the recommended regimen with brief rationale, (2) reasons for discontinuation including overt error, therapy optimization, lack of indication, or other, (3) binary evaluation of the presence of at least one life-threatening recommendation made by the LLM, (4) perceived agreement with the overall medication regimen recommended by the LLM on a 1-5 Likert Scale with 1 being strongly disagree and 5 being strongly agree, and (5) any qualitative comments on perception of the medication regimens. The decision to “continue” or “discontinue” was based on the ground truth which was approved by a majority vote prior to the testing. The presence of a potential life-threatening medication regimen was at the clinician’s discretion. The methods are summarized in Figure 1.

Data Analysis: All statistical analyses were conducted in R version 4.3.1 (2023–06–16). (Team, 2025) The rate of continuation of medications was compared between each LLM using analysis of variance (ANOVA) with a Tukey’s post-hoc test for pairwise comparisons. Identification of life-threatening errors was compared with Chi-squared test for overall comparison. Chi-squared test with Bonferroni adjustment was used for pairwise comparisons. The median rate of agreement of pharmacists with medication regimen on the Likert Scale was assessed with the Kruskal-Wallis test with a post-hoc Dunn’s test with Bonferroni correction for pairwise comparisons. Descriptive analyses were conducted on all variables. Data are reported as mean and standard deviation or median and interquartile range based on parametricity of data.

 Data availability: De-identified case prompts are provided in the Appendix. LLM outputs, clinician item-level ratings and analysis code available upon request.

Use of Generative AI: Generative AI was used as a study instrument but was not used for preparation of this manuscript.

Institutional Review Board: The University of Colorado Institutional Review Board determined this study to be exempt (COMIRB 24–2328).

## Results

The panel consisted of 7 critical care clinicians with board certification in critical care pharmacotherapy. Demographic characteristics are provided in Supplemental Content–Table 1. Patient-case prompts are located in the Supplemental Content–Supplementary Appendix 1.

**TABLE 1:** Pooled rate of medication continuation per LLM.

LLM		Case 1	Case 2	Case 3	Case 4	Case 5	Case 6	Case 7	All cases^a^	p-value
GPT-3.5	Continuation rate, median (IQR)	54.6 (43.2–54.5)	66.7 (45.8–70.8)	66.7 (50–77.8)	55.6 (52.8–66.7)	57.1 (42.9–57.1)	85.7 (64.3–89.3)	61.1 (55.9–70.6)	59.7 (±17.5)	<0.001
	Total medications, n	11	12	18	10	7	14	17	89	
GPT-4	Continuation rate, median (IQR)	64.3 (57.7–67.9)	84.2 (78.9–86.5)	66.7 (55.6–77.8)	57.1 (57.1–60.7)	44.4 (27.8–55.6)	86.7 (80–90)	76.5 (66.7–88.2)	67.3 (±18.1)^a,b^	
	Total medications, n	14	19	18	14	9	15	17	106	
Llama-2-70b	Continuation rate, median (IQR)	60 (50–70)	59 (50–76.2)	72.3 (67.3–79)	33.3 (30–59)	55.6 (44.4–55.6)	63.2 (52.6–68.4)	40 (30–45)	55 (±17.7)^a^	
	Total medications, n	15	21	22	15	9	19	10	111	
Claude-2	Continuation rate, median (IQR)	54.6 (50–59.1)	46.7 (46.7–53.3)	60 (55.2–70)	55.6 (44.4–77.8)	62.5 (37.5–62.5)	41.7 (37.5–62.5)	62.5 (50–62.5)	54.6 (±15.4)^b^	
	Total medications, n	11	15	15	9	8	12	8	78	

LLM: large language model, IQR: interquartile range.

Median percentage of medications that were deemed appropriate for continuation by clinician panel after reviewing LLM-generated medication list.

a, b: rows with matching superscripts are significantly different from each other upon pairwise comparison using Tukey’s test for multiple comparisons (ex. GPT-4, is significantly different compared to both Llama-2–70b and Claude-2). Adjusted p-values for pairwise comparisons using Tukey’s test: GPT-3.5 vs. GPT-4, p = 0.131; GPT-3.5 vs. Llama-2–70b, p = 0.593; GPT-3.5 vs. Claude-2, p = 0.446; ^a^GPT-4, vs. Llama-2–70b, p = 0.003; ^b^GPT-4, vs. Claude-2, p = 0.002; Llama-2–70b vs. Claude-2, p = 0.999.

^a^ All cases reports the mean (±standard deviation) for all clinician reviews of all cases for that LLM (n = 49 [7 cases multiplied by 7 clinician responses]).

As a measure of efficacy, when clinicians evaluated the LLM-generated medication regimens the median percent of medications continued by each clinician was highest for GPT-4 (67.3% ± 18.1%) followed by GPT-3.5 (59.7% ± 17.5%), Llama-2–70b (55% ± 17.7%), and Claude-2 (54.6% ± 15.4%). Upon post-hoc pairwise analysis, GPT-4 had a significantly higher rate of continuation compared to Llama-2–70b (p = 0.003) or Claude-2 (p = 0.002). These results are summarized in Table 1.

For overall agreement with the LLM-generated regimen, the Likert scores were significantly different among LLMs (χ^2^ = 15.93, p = 0.001). Post-hoc pairwise comparison showed that GPT-4 had a significantly higher rate of agreement compared to Llama-2–70b or Claude-2 but other comparisons were not different (see Table 2). Table 3 summarizes rationales for clinician discontinuation of medications in the LLM-generated pharmacotherapy regimen. The median number of medication errors identified by the clinician panel in the pharmacotherapy regimens generated by each LLM were 32, 29, 48, and 34 for GPT-3.5, GPT-4, Llama-2–70b, and Claude-2, respectively, with a total of 224, 222, 325, and 246 errors identified in total for each LLM. Therapy optimization was recommended by the clinician panel for 140 medications in the pharmacotherapy regimens generated by GPT-3.5 and GPT-4, 180 medications in Llama-2–70b, and 138 medications in the pharmacotherapy regimen generated by Claude-2. And Claude-2, while optimization was recommended for 147 medications in the pharmacotherapy regimen generated by Llama-2–70b. Lack of indication was identified by the clinician panel for 58 medication recommendations in GPT-3.5, 68 medication recommendations for GPT-4, 104 medication recommendations for Llama-2–70b, and 64 medication recommendations for Claude-2.

**TABLE 2:** Pooled median Likert scores expressing clinician agreement with each LLM-generated medication regimen.

LLM	Case 1	Case 2	Case 3	Case 4	Case 5	Case 6	Case 7	Overall score, median (IQR)
GPT-3.5	3 (2–3)	1 (1–1.5)	3 (2.5–3.5)	2 (2–3)	1 (1–2)	3 (2.5–4)	1 (1–2)	2 (1–3)
GPT-4	3 (2.5–3.5)	3 (2–3.5)	3 (2.5–3.5)	2 (2–3)	2 (1–2)	3 (2.5–4.5)	2 (1–3)	3 (2–3)^ab^
Llama-2–70b	2 (2–3)	2 (1–2.5)	3 (2–3)	2 (2–3)	1 (1–2)	1 (1–2.5)	1 (1–1)	2 (1–3)^a^
Claude-2	2 (2–2.5)	1 (1–2)	2 (2–2.5)	2 (1–2)	1 (1–2)	1 (1–2.5)	1 (1–1)	2 (1–2)^b^

LLM: large language model, IQR: interquartile range.

a, b: rows with matching superscripts are significantly different from each other upon pairwise comparison using Dunn’s test with Bonferroni correction for multiple comparisons (ex. GPT-4, is significantly different compared to both Llama-2–70b and Claude-2).

Adjusted p-values for pairwise comparisons.

^a^ GPT-4, vs. Llama-2–70b, p = 0.0014.

^b^ GPT-4, vs. Claude-2, p < 0.001; All other pairwise comparisons, non-significant.

**TABLE 3:** Reason for discontinuation of medications by the clinician panel.

Error type	GPT3.5, median (IQR)	GPT4, median (IQR)	Llama-2–70b, median (IQR)	Claude-2, median (IQR)
Overt error	2 (1.5–3)	1 (0–2)	3 (0.5–7)	4 (2–6.5)
Therapy optimization^a^	19 (16.5–19.5)	19 (17–19.5)	22 (21–30.5)	21 (16–24)
Lack of indication	10 (5.5–11)	7 (6.5–13.5)	12 (10–20)	9 (6–12)
Other	1 (1–2)	0 (0–0.5)	1 (0–1.5)	1 (0–3)

For each model the reported median represents the median number of errors reported per clinician across all cases.

LLM, large language model; IQR, interquartile range.

^a^ Therapy optimization would include anything that was deemed not optimal by the clinician panel but not necessarily harmful to the patient (ex. If the LLM, selected a twice daily blood pressure medication as opposed to a simpler once daily regimen, or if it selected an antibiotic that more commonly causes side effects as opposed to a better-tolerated regimen).

As an assessment of safety, the presence of potentially life-threatening recommendations was assessed by clinicians in 57.1% in Claude-2 recommendations followed by 38.8% GPT-3.5 recommendations, 28.6% of Llama-2–70b recommendations, and 16.3% of GPT-4 recommendations. Upon pairwise analysis, GPT-4 had significantly fewer potentially life-threatening errors than GPT-3.5 (p = 0.013) or Claude-2 (p < 0.001) and Llama-2–70b had significantly fewer potentially life-threatening errors than Claude-2 (p = 0.0043) (see Table 4). All other comparisons were non-significantly different. Life-threatening errors per case and a description of those errors are reported in the Supplementary Content- Tables 2, 3.

**TABLE 4:** Medication errors.

LLM	Total errors (across all cases), median (IQR)	Cases with at least 1 clinician reporting a life-threatening error, n (%) N = 7	Rate of life-threatening errors^a^, n (%) N = 49	Chi-square p-value
GPT-3.5	32 (27–34)	7 (100)	19 (38.8)^a^	<0.001
GPT-4	29 (28–34.5)	3 (43.9)	8 (16.3)^b^ ^,^ ^c^	
Llama-2–70b	48 (44–49)	6 (85.7)	14 (28.6)^d^	
Claude-2	34 (33–35)	7 (100)	28 (57.1)^c^ ^,^ ^d^	

a, b, c: rows with matching superscripts are significantly different from each other upon pairwise comparison using Chi-squared test with Bonferroni correction for multiple comparisons (ex. GPT-4, is significantly different compared to both GPT-3.5 and Claude-2).

^a^ percentage is calculated using cases that were assessed as having a potential life threatening error divided by total cases (n = 49).

Adjusted p-values for pairwise comparisons.

^b^ GPT-3.5 vs. GPT-4, p = 0.013, GPT-3.5 vs. Llama-2–70b, p = 0.29, GPT-3.5 vs. Claude-2, p = 0.069, GPT-4, vs. Llama-2–70b, p = 0.15.

^c^ GPT-4, vs. Claude-2, p < 0.001.

^d^ Llama-2–70b vs. Claude-2, p = 0.0043.

## Discussion

In an early evaluation of the ability of LLMs to provide CMM for complex, critically ill patients, a high rate of life-threatening medication recommendations were provided. Of the four LLMs tested GPT-4 had the best performance, demonstrating the highest rates of clinician agreement and lowest rates of life-threatening medical errors. Although the outputs demonstrated contextual grasp of domain-specific content (e.g., correctly matching drugs with doses and routes and matching certain therapies with diseases), LLMs did not consistently evaluate patient specific cases. This study patently supports a stepwise prompting and implementation approach for LLMs in the CMM space.

Using LLMs for medication management has untapped potential given the prolific use of prescription medications and risk for ADEs (Sikora, 2023). However, there are significant challenges that must be overcome. Most LLMs are trained on a widely available corpus (e.g., the Internet), which creates the potential for problems in domains marked by highly technical language or rarely occurring scenarios, as is a hallmark of medical and pharmacy domains (Clusmann et al., 2023; Soroush et al., 2024). Medication use is fraught with errors, so identifying ‘ground truth’ remains a perennial challenge. Additionally, high-quality CMM requires a combination of both recall-based knowledge and application-oriented skills to understand how the individual drug, dose, and formulation interact with the patient, disease, and other medications in a given context to ascertain risk and benefit profiles (Bainum et al., 2024; Branan et al., 2024). Practice-based expertise that encompasses a wide array of relatively rare scenarios is also hard to replicate in datasets. Owing to the challenges as well as potential dangers associated with poor performance, there have been calls for thoughtful evaluation of LLMs prior to use in the healthcare setting (Ayers et al., 2024).

As a key finding of this study, in holistic evaluation clinicians ranked the highest performing LLM as a median 2 out of 5 on level of agreement (i.e., disagree). It is worth noting that given the complexity of the cases and the nuance of clinical practice, there can be differences between a reasonable choice and the best choice. Similarly, “medication error” is a broad term, inclusive of minor oversights with little potential to cause patient harm as well as critical mistakes that can result in significant adverse outcomes). However, our study categorized the reasons why clinical experts discontinued medications recommended by the LLMs and found a high rate of life-threatening pharmacotherapy recommendations, pointing to a concerning knowledge gap for LLMs. For example, in one case with a patient experiencing elevated intracranial pressure, one LLM recommended administering a 250 mL bolus of 23.4% hypertonic saline, a medication that is typically administered as a 30 mL bolus when treating neurologic emergencies: if this had occurred in practice, it would likely have led to significant morbidity and mortality for the patient and notable quality improvement and root cause analysis processes.

There was also a lack of consistency in LLM recommendations across cases with similar features. For example, GPT-3.5 recommended vancomycin in two cases, but different dosing strategies. In one case, it simply recommended vancomycin 1,250 mg x1 with no mention of target trough concentrations, but in another case it recommended 1,250 mg every 12 h with a target trough of 15–20 mg/L. Similarly, GPT-4 had inconsistent recommendations with regards to stress-dose steroids in septic shock. In one case it recommended the addition of steroids for a patient on norepinephrine alone, but in a second case it did not add steroids for a patient on norepinephrine plus vasopressin. This inconsistency in recommendations raises concerns about the background logic being applied by LLMs.

Another observed pattern was a predisposition to continuing medications in the “current medications” content of the case presented to the LLM. This could include continuing a medication without a clear indication for prior-to-admission use (e.g., baclofen in a patient without spasticity) or continuing medications exactly as written in the “current medications” (e.g., “norepinephrine 0.09 mcg/kg/min” rather than norepinephrine titrated to a MAP goal). These patterns give the sense that LLMs are simply transcribing data rather than evaluating the medications on their merits. Other observations included that the LLMs struggled to provide appropriate renal dose adjustments based on patient conditions and committed frequent opioid-related errors (e.g., administering an oral medication intravenously or intravenous opioids to non-intubated patients).

There were some positive observations with regard to data synthesis, particularly with GPT-3.5 and GPT-4. In case 5, GPT-3.5 picked up on “sepsis” in the case and recommended crystalloid 30 mL/kg for the patient in line with best practice guidelines for sepsis management (Evans et al., 2021). Unfortunately, the patient had already received resuscitation, so repeating 30 mL/kg would likely not be indicated. Nonetheless, this observation suggests a stronger ability to collect information from the history of present illness compared to Llama-2–70b or Claude-2. Similarly, in case 4, GPT-4 picked up on “reduced oral intake” in the history of present illness and recommended a fluid bolus “to address dehydration from reduced oral intake”. This represents an impressive ability to collect and synthesize data before making recommendations.

Our methodology was structured to maximize LLM understanding and application of clinical knowledge in the formulation of medication plans (Zhao et al., 2023). By employing reasoning engines (i.e., chain of thought) and one-shot prompting via emphasizing the importance of the in-context demonstration for formatting, we aimed to enhance the models’ ability to process and apply complex medical information. This was further supported by the comparative analysis of the responses across different LLMs, providing insights into their respective capabilities and limitations in medical decision-making tasks. Throughout the study, the effectiveness of the one-shot prompting with in-context learning and the chain-of-thought method was assessed based on the accuracy and clinical relevance of the medication plans generated by the LLMs. The structured approach and comparative analysis offer valuable contributions to the ongoing exploration of the potential of LLMs in healthcare applications, particularly in the context of medication management and treatment planning. The refinement of chain-of-thought (or related concepts like tree-of-thought and graph-of-thought) in combination with zero or few shot learning are rapidly implementable methods even as new medication knowledge and LLM technology progress, which are helpful for keeping such technology up to date. Indeed, this strategy is particularly helpful in healthcare where labeled data (i.e., a dataset with annotated ‘correct’ answers) are scarce and because the prompts support in-context learning, which can strengthen and accelerate the exhaustive fine-tuning process (Ma et al., 2024; Guan et al., 2023). Reasoning engines break up problems into steps from which logical inferences can be made. Our team has shown that zero- and few-shot learning can contribute to dealing with unseen scenarios that lack training datasets, including a new abductive reasoning method via natural language processing (Zhong et al., 2025).

Reasoning engines are useful because they reduce hallucinations and support assessment for logical or training gaps (Holmes et al., 2023; Wei et al., 2022). This structured approach to reasoning can be particularly beneficial in capturing the nuances of clinical decision-making. This study used a one-shot prompting approach in which each model was shown an example case that included a complete “ground truth” medication plan before generating new responses. The exemplar was designed to illustrate how outputs should be structured and reasoned through, not to provide clinical content for reuse. Nevertheless, this setup introduces a potential for in-context leakage: the models could have inferred therapeutic logic or stylistic patterns from the exemplar rather than developing their own reasoning independently. Although the exemplar and test cases involved different patients and clinical details, some overlap in themes (such as sepsis or shock management) may have subtly influenced model outputs. Recognizing this trade-off is important. The exemplar likely improved consistency and formatting across models but may have partially guided their clinical reasoning. Future research could reduce this risk by randomizing or rotating exemplars, using multiple independent examples, or adopting a zero-shot design to isolate genuine model reasoning and generalizability.

This evaluation assesses the ability of LLMs to manage complex medication regimens, with strengths including the establishment of a clinically valid ground truth and inclusion of a diverse clinician panel. However, some limitations exist including that the LLM was not provided all information generally available in the electronic health record and the LLMs were tested on a small number of cases which had similarities throughout and lacked repeating trials to evaluate consistency of model performance. Future analyses would benefit from repeated prompting as well as sensitivity analyses with different model settings. Additionally, the LLMs used were not specifically designed for healthcare-specific assessments, so they likely lacked prior training in these areas. Our analysis was intended to sample LLM capability with different complex cases in critical care, but we recognize that differences in cases (sepsis vs. stroke) may account for some of the variability. However, this proof-of-concept analysis was not designed to explore that component. Additionally, at the time of testing, the LLMs selected were the most up-to-date LLMs available on the market. We recognize that newer models have since been release; however, the latest work suggests that while these models have improved computing capacity, human alignment and domain specific testing remain important.

Ground truth is difficult to establish, as it does still require some aspect of clinical acumen: it is important that future evaluations consider how to account for stylistic variation that is within the confines of evidence-based medicine and not truly reflective of LLM performance. Clinicians may have different opinions on error assessment and adverse event likelihood that may have led to heterogeneity in the “ground truth” determination: this is particularly true in critical care, which observes practice variation given clinical uncertainty in the treatment of various disease states. While our panel attempted to reference guidelines wherever possible, this is a limitation of the study due to practice variation. In clinical scenarios where the guidelines may not be fully applicable to the patient or where there may be several appropriate courses of action, the “ground truth” may be difficult to determine. Though out of scope for this exploratory analysis, establishing how LLMs should act in the setting of clinical uncertainty (i.e., when the ground truth is unknown) is an essential step for their clinical use. In this case, our panel expected to the LLMs to make recommendations that do not overtly cause harm (e.g., high doses of potassium in the setting of renal failure leading to life-threatening arrythmias), to make use of available guidelines whenever available, and to treat the conditions stated in the cases (e.g., antibiotics for sepsis). There is more recent work with LLMs teaching them to say “I do not know,” which may also be a future expectation (Zhang et al., 2024). Notably, the criteria used for evaluating these LLM-generated treatment plans is not standardized and involved human review (instead of automation). Objective, standardized, and ideally automated means of establishing clinical acceptance criteria and performance benchmarking for clinical NLP is an essential area for future development. Indeed, the FDA’s recent viewpoint in JAMA specifically stated that industry and other stakeholders must improve quality assurance and evaluation of artificial intelligence so that there can be consistency and rigor in the critique of artificial intelligence studies (Warraich et al., 2025).

Despite the limitations of this proof-of-concept analysis, findings suggest that available training and fine-tuning methods may support the use of LLMs for treatment selection. The pipeline necessary to develop LLMs to assist with CMM will likely include a thoughtful integration of domain-specific demonstrations including prompt engineering and real-life human feedback and direct preference optimization combined with infrastructure that allows for continual updates as medication knowledge expands. Though these undertakings are time- and resource-intensive, the potential shown here supports future investigations.

## Conclusion

Using present LLMs as a clinical support tool warrants caution, as without thoughtful human interaction, generated recommendations could cause overt harm. However, there is potential for specifically engineered LLMs tailored for medication management given a thoughtful training and fine-tuning paradigm and appropriate clinical benchmarking. Further development is necessary before LLMs can be reliably used as a clinical support tool given their underperformance in this analysis.

## Data Availability

The raw data supporting the conclusions of this article will be made available by the authors, without undue reservation.
