# Supplementary material for: Large language models management of complex medication regimens: a case-based evaluation
Source: Front Pharmacol. 2025 Nov 24;16:1514445. doi: 10.3389/fphar.2025.1514445 (PMC12682882; doi:10.3389/fphar.2025.1514445)
Supplement: Supplementary file 1 [file Supplementaryfile1.docx]

**Supplemental Content**

**Supplemental Table 1.** Demographic features of clinician panel

**Supplemental Table 2**. Life Threatening Errors Rates

**Supplemental Table 3**. Life Threatening Errors Descriptions

**Appendix I.** Patient Case Prompts

**Supplemental Table 1. Demographic features of clinician panel**

|  | Gender | Years in practice | Board certification in critical care pharmacotherapy | Post-graduate residency training in critical care | Geographic region |
| --- | --- | --- | --- | --- | --- |
| 1 | M | 2 | Yes | Yes | Southeast |
| 2 | F | 1 | Yes | Yes | Midwest |
| 3 | M | 11 | Yes | Yes | Northwestern |
| 4 | F | 12 | Yes | Yes | Midwest |
| 5 | F | 3 | Yes | Yes | Southeast |
| 6 | F | 7 | Yes | Yes | Southeast |
| 7 | M | 4 | Yes | Yes | Southeast |

**Supplemental Table 2. Life Threatening Errors Rates**

|  | **Rate of clinicians deeming the LLM response to have a potentially life-threatening error, n (%)** | | | | | | | |  |
| --- | --- | --- | --- | --- | --- | --- | --- | --- | --- |
| **LLM** | **Case 1** | **Case 2** | **Case 3** | **Case 4** | **Case 5** | **Case 6** | **Case 7** | **All Cases** | **ANOVA**  **p-value** |
| GPT-3.5 | 1 (14.3) | 7 (100) | 1 (14.3) | 2 (28.6) | 5 (71.4) | 1 (14.3) | 2 (28.6) | 19 (38.8) | 0.002 |
| GPT-4 | 0 | 0 | 0 | 0 | 4 (57.1) | 1 (14.3) | 3 (42.9) | 8 (16.3) |  |
| Llama-2-70b | 0 | 1 (14.3) | 1 (14.3) | 1 (14.3) | 4 (57.1) | 3 (42.9) | 4 (57.1) | 14 (28.6) |  |
| Claude-2 | 1 (14.3) | 6 (85.7) | 1 (14.3) | 5 (71.4) | 4 (57.1) | 4 (57.1) | 7 (100) | 28 (57.1) |  |

LLM: large language model, IQR: interquartile range

**Supplemental Table 3. Life Threatening Errors**

| Case | Error | LLM(s) |
| --- | --- | --- |
| 1 | No life threatening errors identified |  |
| 2 | Inappropriate antibiotic coverage lacking *Pseudomonal* coverage in a patient growing gram-negative rods with likely septic shock | GPT 3.5, Claude-2 |
| 3 | Increasing nicardipine drip when blood pressures were in normal range may increase risk of hypertensive event | Claude-2 |
| 4 | Missing home antiepileptic medication and increasing risk of seizure | Claude-2 |
| 5 | Administration of an additional 3 liters of fluid resuscitation even though the patient had already received fluid resuscitation increasing risk of volume overload and prolonged mechanical ventilation | Llama-2-70b, GPT4, GPT3.5 |
|  | Not treating acute gastrointestinal bleed or hepatic encephalopathy, increasing risk for death | GPT3.5, Claude-2, Llama-2-70b |
|  | Piperacillin/tazobactam dosing too high for patient’s renal function, increasing risk of seizures | Claude-2 |
|  | Propofol initiated, even though patient is already oversedated, increasing risk of delirium, prolonged ICU stay, and prolonged mechanical ventilation | Claude-2 |
|  | Cefepime dosing too high for patient’s renal function, increasing risk of cefepime-induced neurotoxicity, including seizures | Llama-2-70b, GPT4, GPT 3.5 |
| 6 | Fluid resuscitation with lactated ringers not indicated and lactated ringers is an inappropriate choice given increased intracranial pressure requiring hypertonic saline | Llama-2-70b |
|  | Vasopressor added to a patient who is not hypertensive, increasing risk of stroke and hematoma expansion | Llama-2-70b |
|  | 250milliliters of 23.4% sodium chloride is an extremely high dose, an appropriate dose would be 250milliliters of 3% saline for intracranial pressure, increasing risk of hypernatremia, seizures, and death | Claude-2 |
| 7 | Enoxaparin continued with active hemorrhage and not confirmation of hematoma/hemorrhage stability | GPT4 |
|  | Sedative infusions stopped even though patient has uncontrolled intracranial pressure which may be alleviated with sedative infusions, thus increasing risk of brain damage and herniation | Claude-2, Llama-2-70b |
|  | Fluid resuscitation with lactated ringers not indicated and lactated ringers is an inappropriate choice given increased intracranial pressure requiring hypertonic saline | Llama-2-70b |
|  | Midazolam sedative dose extremely high, risking increased duration of mechanical ventilation and delirium | Llama-2-70b, GPT4, GPT 3.5 |
|  | 250milliliters of 23.4% sodium chloride is an extremely high dose, an appropriate dose would be 250milliliters of 3% saline for intracranial pressure, increasing risk of hypernatremia, seizures, and death | Claude-2 |
|  | Extremely high rate of sodium chloride maintenance fluids, increasing risk of volume overload and increased duration of mechanical ventilation | GPT3.5 |
|  | Dual beta lactams (cefepime and ceftriaxone) with inappropriately high ceftriaxone dose, increasing risk of seizures | GPT 3.5 |

**Appendix I. Patient Case Prompts**

## #1 Patient Case

**H&P Note:** This is an 85-year-old male history of spinal cord injury gastroesophageal reflux disease nonverbal who presents today for vomiting abdominal pain. His urinalysis is positive for blood, nitrates, moderate leukocyte esterase, too numerous to count white blood cells, and 4+ bacteria, although patient does have a chronic Foley catheter with unknown timing of last exchange. Urology was consulted and exchanged foley in the emergency department. CT showed large, formed stool ball in the rectum large stool burden in the distal sigmoid colon suggestive of severe constipation as well as some thickening suggestive of stercoral colitis. Surgery was consulted and attempted disimpaction at bedside but were unsuccessful. Vitals were notable for significant hypotension requiring initiation of norepinephrine after 2 liters of fluids. He was given a dose of vancomycin, ceftriaxone, and metronidazole and admitted to the medical intensive care unit for further workup and management. The patient has not had a bowel movement in several weeks, is cachectic appearing, and is refusing enteral feeding.

**Vital Signs**

MAP: 56 - 71

SBP: 97 - 148

HR: 64 - 103

RR: 16 - 28

Temperature: 34.4-38.1

Non-intubated, saturating from 92 to 100% on room air

**Laboratory Values:**

Sodium: 135

Potassium: 3.9-4.7 (decreasing)

Chloride: 104-109

CO2: 13-14

Glucose: 55 - 106

Blood urea nitrogen: 42-46

Creatinine: 2.33 – 1.93 (decreasing)

Magnesium: 1.7

Phosphorous: 5.5 – 3.6 (decreasing)

Calcium: 7.8 – 8.1

Other relevant labs

WBC: 12.1 – 24.9 (increasing)

Hb: 9.2

Plt: 302

Albumin: 2.7

MRSA/MSSA PCR: positive

Blood cultures and urine cultures negative to date (drawn 5/6, 5/7)

Vancomycin level: 8 mcg/mL

**Home Medication List:**

Aspirin 81 mg daily

Pantoprazole 40 mg daily

Tizanidine 2 mg Q8H

**Current Medications:**

Acetaminophen 650 mg q4h PRN pain 1-3

Ceftriaxone 1000 mg IV q24

Dextrose 5% 0.9% NaCl 50 mL/hr

D50 25 mL PRN

D50 50 mL PRN

Glucagon 1 mg PRN

Glucose tab 16 gm PO PRN

Glucose 40% gel 1 application PRN

Heparin 5000 unit q8h

Hydrocortisone 50 mg IV q6h

Levothyroxine 75 mcg daily

Magnesium hydroxide 30 mL PO BID PRN constipation

Metronidazole 500 mg IV q8h

Multivitamin with minerals 1 tab PO Daily

Norepinephrine continuous infusion 0.09 mcg/kg/hr (maximum was yesterday at 0.3 mcg/kg/hr)

Pantoprazole 40 mg PO BID

Polyethylene glycol 17 gm PO Qday

Polyethylene glycol 17 gm PO BID

Senna 8.6 mg QHS

Vancomycin PRN dosing per pharmacy

Vancomycin 1250 mg IV x 1

**Ground Truth:**

Acetaminophen 650 mg q4h PRN pain 1-3

**Piperacillin-tazobactam 4.5 gm IV Q8H**

Dextrose 5% 0.9% NaCl 50 mL/hr

D50 25 mL PRN

D50 50 mL PRN

Glucagon 1 mg PRN

Glucose tab 16 gm PO PRN

Glucose 40% gel 1 application PRN

Heparin 5000 unit q8h

Hydrocortisone 50 mg IV q6h

Levothyroxine 75 mcg daily

Multivitamin with minerals 1 tab PO Daily

Norepinephrine continuous infusion 0.09 mcg/kg/hr (maximum was yesterday at 0.3 mcg/kg/hr)

Pantoprazole 40 mg PO BID

**Polyethylene glycol 17 gm PO Q8H**

Senna 8.6 mg QHS

**Commentary:**

- Would consolidate vanc, metronidazole, ceftriaxone to zosyn to cover for enterococcus and other enteric pathogens.
- MRSA PCR positive but not worried about pneumonia so ok d/c’ing vancomycin if enterococcus covered with penicillin
- Consolidate two PEG orders to a Q8H order
- Patients whole reason for being here is stool ball and IAI potential– would probably escalate with more laxatives i.e. lactulose and get rid of PRNs, should be physician talking about it

## #2

**H&P Note:**

This is an 85-year-old male history of spinal cord injury gastroesophageal reflux disease nonverbal who presents today for vomiting abdominal pain. His urinalysis is positive for blood, nitrates, moderate leukocyte esterase, too numerous to count white blood cells, and 4+ bacteria, although patient does have a chronic Foley catheter with unknown timing of last exchange. Urology was consulted and exchanged foley in the emergency department. CT showed large, formed stool ball in the rectum large stool burden in the distal sigmoid colon suggestive of severe constipation as well as some thickening suggestive of stercoral colitis. Surgery was consulted and attempted disimpaction at bedside but were unsuccessful. Patient’s caregiver reports difficulty breathing developing over the past 3 days and subjective fever. Vitals were notable for significant hypotension requiring initiation of norepinephrine after 2 liters of fluids. He was given a dose of vancomycin and metronidazole and admitted to the medical intensive care unit for further workup and management. The patient has not had a bowel movement in several weeks, is cachectic appearing, and is refusing enteral feeding.

**Vital Signs**

MAP: 40 - 68

SBP: 97 - 110

HR: 96 - 112

RR: 22 - 28

Temperature: 39.1

On 6 L nasal cannula saturating 90-95%

**Laboratory Values:**

Sodium: 135

Potassium: 3.9-4.7 (decreasing)

Chloride: 104-109

CO2: 10

Glucose: 55 - 106

Blood urea nitrogen: 42-46

Creatinine: 2.4 (decreasing)

Magnesium: 1.7

Phosphorous: 3.6 – 5.5 (increasing)

Calcium: 7.8 – 8.1

**Other relevant labs**

WBC: 12.1 – 24.9 (increasing)

Hb: 9.2

Plt: 302

Albumin: 2.7

MRSA/MSSA PCR: positive

Blood cultures and urine cultures negative to date (drawn 5/6, 5/7)

Sputum culture: non-lactose fermenting gram negative rods

Vancomycin level: 8 mcg/mL

**Home Medication List:**

Aspirin 81 mg daily

Pantoprazole 40 mg PO BID

Tizanidine 2 mg Q8H

**Current Medications:**

Acetaminophen 650 mg q4h PRN pain 1-3

Ceftriaxone 1000 mg IV q24

Dextrose 5% 0.9% NaCl 50 mL/hr

D50 25 mL PRN

D50 50 mL PRN

Glucagon 1 mg PRN

Glucose tab 16 gm PO PRN

Glucose 40% gel 1 application PRN

Levothyroxine 75 mcg daily

Magnesium hydroxide 30 mL PO BID PRN constipation

Multivitamin with minerals 1 tab PO Daily

Norepinephrine continuous infusion 0.26 mcg/kg/hr

Polyethylene glycol 17 gm PO Qday

Polyethylene glycol 17 gm PO BID

Senna 8.6 mg QHS

**Ground Truth:**

Acetaminophen 650 mg q4h PRN pain 1-3

**Piperacillin-tazobactam 4.5 gm IV Q8H**

Dextrose 5% 0.9% NaCl 50 mL/hr

D50 25 mL PRN

D50 50 mL PRN

Glucagon 1 mg PRN

Glucose tab 16 gm PO PRN

Glucose 40% gel 1 application PRN

**Heparin 5000 unit q8h**

**Hydrocortisone 50 mg IV q6h**

Levothyroxine 75 mcg daily

Multivitamin with minerals 1 tab PO Daily

Norepinephrine continuous infusion 0.26

**Vasopressin 2.5 unit/hr**

**Pantoprazole 40 mg PO BID**

**Polyethylene glycol 17 gm PO Q8H**

Senna 8.6 mg QHS

Vancomycin pulse dose

**Commentary:**

Would add Zosyn for GRN NLF coverage in sputum since patient is experiencing respiratory symptoms. Also covers anaerobes for IAI process

Need an order for vancomycin, only received one dose in ED and not reordered / level = 8, MRSA PCR positive so reasonable to keep

Consolidate two PEG orders to a Q8H order

Norepinephrine 0.26 mcg/kg/min, need to add second vasopressor to minimize and add hydrocortisone shock dose

Need to add DVT prophylaxis

Need to add pantoprazole 40 mg BID because home med but also technically due to shock he would qualify for GI ppx

## #3

**H&P Note:**

31 year old male with history of end stage renal disease on hemodialysis, hypertension, diabetes, syringomyelia with functional paraplegia presenting with respiratory distress. Patient was intubate in emergency department.

**Vital Signs:**

MAP: 90-134

SBP: 110-155

HR: 70-82

RR: 11-20

Temperature: 35.8-37.6

**Laboratory Values:**

Sodium: 135

Potassium:3.6

Chloride: 91

CO2: 30

Glucose:272

Blood urea nitrogen: 25

Creatinine: 3.81

Magnesium: 2

Phosphorous: 3

Calcium:9.4

BNP 2700

MRSA PCR +

**ABG + basic ventilator settings:**

pH: 7.64

PaCO2:

PaO2:164

HCO3: 32.5

Mode:PRVC

Rate: 14

Tidal volume: 500

Pressure: 8

**Other relevant elements:**

Recent admission to hospital (<3 months ago, with receipt of IV antibiotics)

History of klebsiella pneumo in urine

RASS -3

**Home Medication List:**

Amlodipine 10mg PO daily

Aspirin 81mg daily

Calcitriol 0.5mcg PO daily

Carvedilol 25mg PO BID

Clotrimazole ointment BID

Famotidine 20mg BID

Ferrous sulfate 325mg daily

Folic acid 1mg daily

Hydralazine 25mg po q8h

Hydrocortisone ointment bid

Glargine 18 units SQ QHS lispro 10units TIDAC

Loperamide 2mg PO Q6h

Rosuvastatin 5mg daily

Sevelamer 800mg TIDWM

**Today’s MAR**

Amlodipine 10mg PO daily

Aspirin 81mg daily

Baclofen 10mg Q8H

Carvedilol 12.5mg q12h

Cefepime 1g q24h

Chlorhexidine 15ml PO BID

Famotidine 20mg daily

SQ heparin 5000 unit q8h

Hydralazine 50mg q8h

Insulin glargine 15 units

Insulin lispro 7 units q4h

Sliding scale insulin

Losartan 50mg daily

Vancomycin pulse dosing

PRN fentanyl 50mcg

Dexmedetomidine 1.5mcg/kg/hr

Fentanyl 3.5mcg/kg/hr

Nicardipine 1mg/hr

**Ground Truth:**

Amlodipine 10mg PO daily

Aspirin 81mg daily

Folic acid 1mg daily

Carvedilol 25mg q12h

Cefepime 1g q24h

Chlorhexidine 15ml PO BID

Famotidine 20mg daily

SQ heparin 5000 unit q8h

Hydralazine 25mg q8h

Insulin glargine 18 units

Insulin lispro 10 units TIDAC

Rosuvastatin 5mg daily

Sliding scale insulin

Losartan 50mg daily

Vancomycin pulse dosing

PRN fentanyl 50mcg

Dexmedetomidine 1.5mcg/kg/hr

Fentanyl 2mcg/kg/hr

**Commentary:**

D/c nicardipine – basically off already, dose too low to matter

Increase insulin to home regimen – blood glucose >180 (NICE SUGAR trial)

Add home rosuvastatin – good to start home meds if not contraindicated, could probably cite some statin trial here

Add folic acid – good to start home meds if not contraindicated

Increase carvedilol to 25mg BID and try to decrease hydralazine to home dosing – carvedilol is better for blood pressure than hydralazine (see HTN guidelines)

Decrease fentanyl infusion to 2mcg/kg/min to target RASS 0 to -2 – PADIS guidelines, goal RASS 0 to -2

Dc baclofen – unclear indication

## #4

**H&P Note:** 58 year old female presenting as a code stroke after falling out of her wheelchair and hitting her head. Stroke workup has been negative and neurology has recommended workup for metabolic encephalopathy. She has had reduced PO intake for the past 10 days after being diagnosed with a urinary tract infection and started nitrofurantoin and ciprofloxacin.

**Vital Signs:**

MAP: 55-60

SBP: 94-138

HR: 79-113

RR: 16-24

Temperature: 37.1-38.7

**Laboratory Values:**

Sodium: 134

Potassium:3.6

Chloride: 99

CO2: 25

Glucose: 137

Blood urea nitrogen: 16

Creatinine: 0.55

Magnesium:

Phosphorous:

Calcium:

WBC: 13.7

**Other relevant elements:**

Blood cultures preliminarily identified as enterococcus faecalis

Penicillin allergy – angioedema – severe

**Home Medication List:**

Acetaminophen 975mg PO q6h

Aspirin 81mg daily

Atorvastatin 40mg daily

Ergocalciferol 50000 units weekly

Lacosamide 50mg BID

Levetiracetam 1500mg BID

Melatonin 6mg QHS

Multivitamin daily

Oxycodone 5mg PO q4h PRN pain

Sitagliptin 100mg PO daily

Nitrofurantoin 100mg BID

Ciprofloxacin 250mg BID

Citalopram 20mg daily

**Today’s MAR:**

Atorvastatin 40mg QPM

Aspirin 81mg daily

Cefepime 2g q8h IV

Citalopram 20mg daily

Famotidine 20mg PO BID

SQ heparin 5000 unit q8h

Lacosamide 100mg BID

LR 1500ml bolus x 1

Levetiracetam 500mg BID

Metronidazole 500mg q8h

Vancomycin 1250mg q8h

Norepinephrine 0.06mcg/kg/min

Ground Truth:

Atorvastatin 40mg QPM

Aspirin 81mg daily

Citalopram 20mg daily

Famotidine 20mg PO BID

SQ heparin 5000 unit q8h

Lacosamide 50mg BID

LR 1500ml bolus x 1

Levetiracetam 1500mg BID

Vancomycin 1250mg q8h

Norepinephrine 0.06mcg/kg/min

Ergocalciferol 50000 units weekly

Multivitamin daily

Sliding scale insulin SQ q6h

Commentary:

D/c metronidazole – does not cover enterococcus faecalis

d/c cefepime – does not cover enterococcus faecalis

Increase levetiracetam to home dosing – do not want patient to have a seizure

Decrease lacosamide to home dosing – no reason to give higher dose if patient was previously controlled on lower dose

Resume ergocalciferol – good to start home meds if not contraindicated

Resume multivitamin – good to start home meds if not contraindicated

Start sliding scale insulin – on sitagliptin at home

Assess volume status – surviving sepsis campaign

## #5

**H&P Note:** 47 y.o. female with opiate abuse, tobacco use, esophageal varices s/p banding (01/2023) and decompensated cirrhosis without ongoing hepatology care who presents from OSH ED with confusion/somnolence, hematemesis x 1, generalized abdominal pain, and poor PO intake for the past 3 days. Was hypotensive with MAP of 46 requiring pressor support and intubation. Transferring to UNC MICU for work-up of newly AKI and shock likely secondary to sepsis vs hypovolemic in setting of AMS, UGIB, and abdominal pain likely 2/2 decompensated cirrhosis.

**Objective/Vital Signs:**

Ht: 165.5 cm

Wt 75 kg

MAP: 46

SBP: 73

HR: 107

RR: 23

Temperature: 38.7 C

**Laboratory Values:**

Sodium: 125

Potassium: 5.2

Chloride: 96

CO2: 21

Glucose: 120

Blood urea nitrogen: 42

Creatinine: 3.61

Magnesium: 2.8

Phosphorous: 6.0

Calcium: 10.0

Lactate 2.1

WBC 23.4

Hgb 10.8

PLT 150

**ABG + ventilator settings:**

pH: 7.32

PaCO2: 40

PaO2: 94

HCO3: 20.7

Mode: Volume Control

Rate: 16

Tidal volume: 450

PEEP: 5

FiO2: 60%

CXR with RLL infiltrate

Lower respiratory culture growing Staph Aureus, positive MRSA Screen

RASS -5

**Today’s MAR**

Norepinephrine 30 mcg/min

Cefepime 2 g q8h

Daptomycin 500 mg q48 h

Lactated Ringer’s 1000 mL x3 (given)

Propofol 20 mcg/kg/min**Ground Truth:**

Norepinephrine 30 mcg/min

Vasopressin 0.03 units/min

Hydrocortisone 50 mg q6h

Cefepime 1 g q24h

Vancomycin (target 15-20 mg/mL)

Acetaminophen 1000 mg q8h

Oxycodone 5 mg q4h PRN moderate pain

Fentanyl 25 mcg q2h PRN Severe Pain

**Commentary:**

Hydrocortisone: The patient is still requiring two vasopressors at high doses despite adequate volume resuscitation. Guidelines and literature would support addition of stress dose steroids

Vasopressin: Standard of care is addition of vasopressin to norepinephrine for catecholamine-sparing effects

Cefepime: Dose reduction to 1 g q24h with an estimated CrCL of ~20 mL/min

Vancomycin: no risk factor for VRE; Given + MRSA screen and growth of Staph aureus from lower respiratory culture, MRSA pneumonia coverage is indicated. Given that daptomycin is inactivated by pulmonary surfactants, recommend switching to vancomycin

APAP: Scheduled APAP for pain control

## #6

This person was a 50 year old male presenting as a level 1 trauma after a motorcycle collision in which he was the unhelmeted driver that reportedly struck a tree and a stop sign. He had a decreased Glasgow coma scale on scene and upon arrival to emergency department showed a Glasgow coma scale of 6. Unable to obtain any additional history from patient. Patient intubated in the emergency department for airway protection. Of note, he has a history of motor vehicle collision yesterday and motorcycle collision in 2008 as well. At that time no significant past medical history was noted. Imaging significant for subdural hematoma, subarachnoid hemorrhage, intraparenchymal hemorrhage, bilateral temporal bone fractures, right Cervical 7 transverse process fracture, right 2nd/3rd rib fracture, right upper lung and right middle lung contusions, trace right pneumothorax, right LeFort III pattern fracture, right frontal bone fracture, dissections of the right common carotid and left internal carotid arteries. Patient admitted to trauma intensive care unit for every 1 hour neurologic checks and mechanical ventilation. Extraventricular drain was placed by neurosurgery and facial lacerations were repaired by oral maxillofacial surgery.

Interval: Night team reported patient had a bloody drainage from his nose. Ears nose and throat doctors were engaged to assess patients continued bloody nose. Intracranial pressures ranged from 12-40 mmHg. Patient spiked intracranial pressures after a intravenous fentanyl push. Patient self corrects intracranial pressures within a few minutes. 23% hypertonic saline was ordered and on standby. Systolic blood pressures have been 130-180 mmHg. Patient responds well to labetalol.

**Vital Signs:**

MAP: 78 – 101

SBP: 134- 180

HR: 57 – 82

RR: 16 (set on vent) 21 actual

Temperature: 37.9 – 38.3 deg C

**Laboratory Values:**

Sodium: 141

Potassium: 4.1

Chloride: 107

CO2: 24

Glucose: 159

Blood urea nitrogen: 28

Creatinine: 0.57

Magnesium: 2

Phosphorous: 3.5

Calcium: 8.4

**ABG + basic ventilator settings:**

pH: 7.47

PaCO2: 36.1

PaO2:93

HCO3: 26.2

Mode: PRVC

Rate: Set 14, actual 21

Tidal volume: 450

Pressure: peak inspiratory pressure 31, mean airway 16

**Other relevant elements:**

From brocheoalveolar lavage 4 days prior

Colony count of >100,000 CFU/mL Serratia marcescens sensitive to cefepime and sensitive to ceftriaxone and resistant to cefazolin

Colony count of >100,000 CFU/mL Staphylococcus aureus methicillin-susceptible

Chest x-ray: Demonstrates improvement in aeration.

Intracranial pressures have range from 4 to 32 mmHg with values over 20 occurring for only a few minutes then self-resolving to values < 20 mmHg.

**Home Medication List**

Ultracet, 1 tab, PO, Q4HR, 1 refills

**Medication Regimen:**

acetaminophen oral suspension 975 mg by mouth every 6 hours

Aspirin chewable 81 mg by mouth every day

Bisacodyl rectal suppository per rectum every 48 hours

Cefepime 2 g intravenously every 8 hours

Chlorhexidine topical 15 mL buccal every 12 hours

Docusate 100 mg oral liquid by mouth twice daily

Enoxaparin 40 g subcutaneously every 12 hours

Famotidine 20 mg tablet by mouth every 12 hours

Methocarbamol 500 mg tablet by mouth every 8 hours

Saline flush for central line 20 mL injection every 12 hours

Senna 8.6 mg by mouth twice daily

Sodium chloride 23.4 % 120 milliequivalents / 30 mL intravenously once

Fentanyl 50 micrograms administered IV once as part of a as needed medication

Hydralazine 10 mg administered IV once as part of a as needed medication

Fentanyl infusion 1.5 mcg/kg/hr (4.94 ml/hr)

Midazolam infusion 8.5 mg/hr (8.5 ml/hr)

Sodium chloride 0.9% infusion 100 ml/hour

Propofol infusion 40 mcg/kg/min 15.82 ml/hour

**Ground Truth:**

Discontinue cefepime and start ceftriaxone as ceftriaxone covers both Serratia marcescens and Staphylococcus aureus methicillin-susceptible. Ceftriaxone is more narrow spectrum than cefepime and therefore does not need to be used in the absence of a resistant bacteria in this circumstance. Also I would decrease sedation as intracranial pressures have been stable and the patient can continue to wean his high dose midazolam infusion as it has been on the same rate for 24 hours with minimal intracranial pressure elevations.

acetaminophen oral suspension 975 mg by mouth every 6 hours

Aspirin chewable 81 mg by mouth every day

Bisacodyl rectal suppository per rectum every 48 hours

Ceftriaxone 2 g intravenously every 24 hours

Chlorhexidine topical 15 mL buccal every 12 hours

Docusate 100 mg oral liquid by mouth twice daily

Enoxaparin 40 g subcutaneously every 12 hours

Famotidine 20 mg tablet by mouth every 12 hours

Methocarbamol 500 mg tablet by mouth every 8 hours

Saline flush for central line 20 mL injection every 12 hours

Senna 8.6 mg by mouth twice daily

Sodium chloride 23.4 % 120 milliequivalents / 30 mL intravenously once

Fentanyl 50 micrograms administered IV once as part of a as needed medication

Hydralazine 10 mg administered IV once as part of a as needed medication

Drips:

Fentanyl infusion 1.5 mcg/kg/hr (4.94 ml/hr)

Midazolam infusion 7.5 mg/hr (7.5 ml/hr)

Sodium chloride 0.9% infusion 100 ml/hour

Propofol infusion 40 mcg/kg/min 15.82 ml/hour

## #7

This person was a 50 year old male presenting as a level 1 trauma after a motorcycle collision in which he was the unhelmeted driver that reportedly struck a tree and a stop sign. He had a decreased Glasgow coma scale on scene and upon arrival to emergency department showed a Glasgow coma scale of 6. Unable to obtain any additional history from patient. Patient intubated in the emergency department for airway protection. Of note, he has a history of motor vehicle collision yesterday and motorcycle collision in 2008 as well. At that time no significant past medical history was noted. Imaging significant for subdural hematoma, subarachnoid hemorrhage, intraparenchymal hemorrhage, bilateral temporal bone fractures, right Cervical 7 transverse process fracture, right 2nd/3rd rib fracture, right upper lung and right middle lung contusions, trace right pneumothorax, right LeFort III pattern fracture, right frontal bone fracture, dissections of the right common carotid and left internal carotid arteries. Patient admitted to trauma intensive care unit for every 1 hour neurologic checks and mechanical ventilation. Extraventricular drain was placed by neurosurgery and facial lacerations were repaired by oral maxillofacial surgery.

Interval: Night team reported patient had a bloody drainage from his nose. Ears nose and throat doctors were engaged to assess patients continued bloody nose. Intracranial pressures ranged from 12-40 mmHg. Patient spiked intracranial pressures after a intravenous fentanyl push. Patient self corrects intracranial pressures within a few minutes. 23% hypertonic saline was ordered and on standby. Systolic blood pressures have been 130-180 mmHg. Patient responds well to labetalol.

**Vital Signs:**

MAP: 78 – 101

SBP: 134- 180

HR: 57 – 82

RR: 16 (set on vent) 21 actual

Temperature: 37.9 – 38.3 deg C

**Laboratory Values:**

Sodium: 141

Potassium: 4.1

Chloride: 107

CO2: 24

Glucose: 159

Blood urea nitrogen: 28

Creatinine: 0.57

Magnesium: 2

Phosphorous: 3.5

Calcium: 8.4

**ABG + basic ventilator settings:**

pH: 7.47

PaCO2: 36.1

PaO2:93

HCO3: 26.2

Mode: PRVC

Rate: Set 14, actual 21

Tidal volume: 450

Pressure: peak inspiratory pressure 31, mean airway 16

**Other relevant elements;**

From brocheoalveolar lavage 4 days prior

Colony count of >100,000 CFU/mL Serratia marcescens sensitive to cefepime and sensitive to ceftriaxone and resistant to cefazolin

Colony count of >100,000 CFU/mL Staphylococcus aureus methicillin-susceptible

Chest x-ray: Demonstrates improvement in aeration.

Intracranial pressures have range from 4 to 32 mmHg with values over 20 occurring for only a few minutes then self-resolving to values < 20 mmHg.

**Home Medication List**

Ultracet, 1 tab, PO, Q4HR, 1 refills

**Medication Regimen:**

Cefazolin 1 g intravenously every 24 hours

acetaminophen oral suspension 975 mg by mouth every 6 hours

Aspirin chewable 81 mg by mouth every day

Bisacodyl rectal suppository per rectum every 48 hours

Chlorhexidine topical 15 mL buccal every 12 hours

Docusate 100 mg oral liquid by mouth twice daily

Enoxaparin 40 g subcutaneously every 12 hours

Famotidine 20 mg tablet by mouth every 12 hours

Methocarbamol 500 mg tablet by mouth every 8 hours

Saline flush for central line 20 mL injection every 12 hours

Senna 8.6 mg by mouth twice daily

Sodium chloride 23.4 % 120 milliequivalents / 30 mL intravenously once

Fentanyl 50 micrograms administered IV once as part of a as needed medication

Hydralazine 10 mg administered IV once as part of a as needed medication

Drips:

Fentanyl infusion 1.5 mcg/kg/hr (4.94 ml/hr)

Midazolam infusion 20 mg/hr (20 ml/hr)

Sodium chloride 0.9% infusion 400 ml/hour

Propofol infusion 10 mcg/kg/min 3.96 ml/hour

**Ground Truth:**

Discontinue cefepime and start ceftriaxone as ceftriaxone covers both Serratia marcescens and Staphylococcus aureus methicillin-susceptible. Ceftriaxone is more narrow spectrum than cefepime and therefore does not need to be used in the absence of a resistant bacteria in this circumstance. Also I would decrease sedation as intracranial pressures have been stable and the patient can continue to wean his high dose midazolam infusion as it has been on the same rate for 24 hours with minimal intracranial pressure elevations.

acetaminophen oral suspension 975 mg by mouth every 6 hours

Aspirin chewable 81 mg by mouth every day

Bisacodyl rectal suppository per rectum every 48 hours

Ceftriaxone 2 g intravenously every 24 hours

Chlorhexidine topical 15 mL buccal every 12 hours

Docusate 100 mg oral liquid by mouth twice daily

Enoxaparin 40 g subcutaneously every 12 hours

Famotidine 20 mg tablet by mouth every 12 hours

Methocarbamol 500 mg tablet by mouth every 8 hours

Saline flush for central line 20 mL injection every 12 hours

Senna 8.6 mg by mouth twice daily

Sodium chloride 23.4 % 120 milliequivalents / 30 mL intravenously once

Fentanyl 50 micrograms administered IV once as part of a as needed medication

Hydralazine 10 mg administered IV once as part of a as needed medication

Fentanyl infusion 1.5 mcg/kg/hr (4.94 ml/hr)

Midazolam infusion 7.5 mg/hr (7.5 ml/hr)

Sodium chloride 0.9% infusion 100 ml/hour

Propofol infusion 40 mcg/kg/min 15.82 ml/hour

1. ASHP. Comprehensive medication management, Available from <https://www.ashp.org/advocacy-and-issues/key-issues/other-issues/comprehensive-medication-management?loginreturnUrl=SSOCheckOnly#:~:text=Definition%20of%20CMM%3A%20The%20standard,effective%20for%20the%20medical%20condition>. Accessed 4/15/24,

2. Leape LL, Cullen DJ, Clapp MD, et al. Pharmacist participation on physician rounds and adverse drug events in the intensive care unit. JAMA 1999;3:267-70.

3. Nuckols TK, Smith-Spangler C, Morton SC, et al. The effectiveness of computerized order entry at reducing preventable adverse drug events and medication errors in hospital settings: a systematic review and meta-analysis. Syst Rev 2014;56.

4. Slight SP, Seger DL, Franz C, Wong A, Bates DW. The national cost of adverse drug events resulting from inappropriate medication-related alert overrides in the United States. J Am Med Inform Assoc 2018;9:1183-88.

5. Tariq RA VR, Sinha A, et al. . Medication Dispensing Errors and Prevention. [Updated 2023 May 2]. In: StatPearls [Internet]. Treasure Island (FL): StatPearls Publishing; 2023 Jan-. Available from: <https://www.ncbi.nlm.nih.gov/books/NBK519065/>.

6. Stollings JL, Poyant JO, Groth CM, et al. An International, Multicenter Evaluation of Comprehensive Medication Management by Pharmacists in ICU Recovery Centers. J Intensive Care Med 2023;10:957-65.

7. Holmes J, Liu Z, Zhang L, et al. Evaluating Large Language Models on a Highly-specialized Topic, Radiation Oncology Physics. arXiv 2023.

8. R. R Core Team (2021). R: A language and environment for statistical ## computing. R Foundation for Statistical Computing, Vienna, Austria. <https://www.R-project.org/>. ,

9. Sikora A. Critical Care Pharmacists: A Focus on Horizons. Crit Care Clin 2023;3:503-27.

10. Ayers JW, Desai N, Smith DM. Regulate Artificial Intelligence in Health Care by Prioritizing Patient Outcomes. JAMA 2024;8:639-40.

11. Hawkins WA PR. Cultivating Expert Thinking Skills for Experiential Pharmacy Trainees. . Am J Health Syst Pharm 2024.

12. Branan TN, Darley A, Hawkins WA. How critical is it? Integrating critical care into the pharmacy didactic curriculum. Am J Health Syst Pharm 2024.

13. Lin Zhao LZ, Zihao Wu, Yuzhong Chen, Haixing Dai, Xiaowei Yu, Zhengliang Liu, Tuo Zhang, Xintao Hu, Xi Jiang, Xiang Li, Dajiang Zhu, Dinggang Shen, Tianming Liu. . When Brain-inspired AI Meets AGI. <https://arxivorg/abs/230315935> Accepted by Meta-Radiology 2023.

14. Ma C, Wu Z, Wang J, et al. ImpressionGPT: An Iterative Optimizing Framework for Radiology Report Summarization with ChatGPT. arXiv 2023.

15. Guan Z, Wu Z, Liu Z, et al. CohortGPT: An Enhanced GPT for Participant Recruitment in Clinical Study. arXiv 2023.

16. Jason Wei YT, Rishi Bommasani, Colin Raffel, Barret Zoph, Sebastian Borgeaud, Dani Yogatama, Maarten Bosma, Denny Zhou, Donald Metzler, Ed H. Chi, Tatsunori Hashimoto, Oriol Vinyals, Percy Liang, Jeff Dean, William Fedus. Emergent Abilities of Large Language Models. <https://arxivorg/abs/220607682> 2022.
